# Interventions to improve the appropriate use of polypharmacy in older people: a Cochrane systematic review

**DOI:** 10.1136/bmjopen-2015-009235

**Published:** 2015-12-09

**Authors:** Janine A Cooper, Cathal A Cadogan, Susan M Patterson, Ngaire Kerse, Marie C Bradley, Cristín Ryan, Carmel M Hughes

**Affiliations:** 1 School of Pharmacy, Queen's University Belfast, Belfast, UK; 2 Belfast, UK; 3 Department of General Practice and Primary Health Care, University of Auckland, Auckland, New Zealand

**Keywords:** polypharmacy, aged, systematic review, interventions, GERIATRIC MEDICINE

## Abstract

**Objective:**

To summarise the findings of an updated Cochrane review of interventions aimed at improving the appropriate use of polypharmacy in older people.

**Design:**

Cochrane systematic review. Multiple electronic databases were searched including MEDLINE, EMBASE and the Cochrane Central Register of Controlled Trials (from inception to November 2013). Hand searching of references was also performed. Randomised controlled trials (RCTs), controlled clinical trials, controlled before-and-after studies and interrupted time series analyses reporting on interventions targeting appropriate polypharmacy in older people in any healthcare setting were included if they used a validated measure of prescribing appropriateness. Evidence quality was assessed using the Cochrane risk of bias tool and GRADE (Grades of Recommendation, Assessment, Development and Evaluation).

**Setting:**

All healthcare settings.

**Participants:**

Older people (≥65 years) with ≥1 long-term condition who were receiving polypharmacy (≥4 regular medicines).

**Primary and secondary outcome measures:**

Primary outcomes were the change in prevalence of appropriate polypharmacy and hospital admissions. Medication-related problems (eg, adverse drug reactions), medication adherence and quality of life were included as secondary outcomes.

**Results:**

12 studies were included: 8 RCTs, 2 cluster RCTs and 2 controlled before-and-after studies. 1 study involved computerised decision support and 11 comprised pharmaceutical care approaches across various settings. Appropriateness was measured using validated tools, including the Medication Appropriateness Index, Beers’ criteria and Screening Tool of Older Person's Prescriptions (STOPP)/ Screening Tool to Alert doctors to Right Treatment (START). The interventions demonstrated a reduction in inappropriate prescribing. Evidence of effect on hospital admissions and medication-related problems was conflicting. No differences in health-related quality of life were reported.

**Conclusions:**

The included interventions demonstrated improvements in appropriate polypharmacy based on reductions in inappropriate prescribing. However, it remains unclear if interventions resulted in clinically significant improvements (eg, in terms of hospital admissions). Future intervention studies would benefit from available guidance on intervention development, evaluation and reporting to facilitate replication in clinical practice.

**Strengths and limitations of this study**

- The updated Cochrane review that is summarised in this paper used systematic and rigorous methods to identify, appraise and synthesise available evidence for the effectiveness of interventions aimed at improving appropriate polypharmacy for older patients.
- No language restrictions were placed on the search strategy and no apparent publication bias was detected.
- The included studies were limited by their small sample sizes and poor quality owing to risks of bias, with little opportunity to pool data.
- Despite improvements in appropriate prescribing, it must be noted that assessments were based on surrogate markers of appropriate polypharmacy and the clinical significance of these improvements in terms of other relevant outcomes, for example, hospital admissions, is unclear.
- Several studies focused on reducing the number of medications, rather than improving the overall appropriateness of prescribing, including underprescribing.

## Introduction

The WHO has predicted that the number of older people (conventionally defined as ≥65 years) worldwide will reach 1.5 billion by 2050.^1^ ^2^ This population growth poses significant challenges for healthcare systems, as older people use a disproportionate amount of healthcare resources (eg, medications).^3^ ^4^

Although there is no single agreed definition of the term ‘polypharmacy’,^5^ ^6^ this has been described as the use of four or more medications.^7^ The potential for negative outcomes with the use of multiple medications in older people is well documented (eg, adverse drug events (ADEs), non-adherence, drug interactions).^8^ ^9^ A critical objective that poses considerable challenges for healthcare professionals (HCPs) is to obtain a balance between aggressively treating diseases and avoiding medication-related harm.^10^

Polypharmacy has been identified as the principal determinant of potentially inappropriate prescribing (PIP) in older people.^11^ The term PIP encompasses overprescribing, misprescribing and underprescribing.^12^ Underprescribing is an important clinical issue because patients with polypharmacy have an increased likelihood of not receiving potentially beneficial, clinically indicated medications compared with patients receiving fewer medications.^13^ Accordingly, a range of assessment tools have been developed to identify PIP in older people and to optimise prescribing.^14^

Despite the potential for negative consequences in older patients receiving polypharmacy, there is increasing acceptance that the prescribing of multiple medications can be appropriate, and under certain circumstances, should be encouraged.^15^ ^16^ Thus, polypharmacy can refer to the prescribing of many drugs (appropriately) or too many drugs (inappropriately).^16^ Achieving appropriate polypharmacy involves prescribing the correct drugs under the appropriate circumstances to treat the right diseases. Ensuring appropriate polypharmacy is of considerable importance because PIP is highly prevalent in older people and has considerable cost implications for healthcare systems.^11^ ^17^

The updated Cochrane review that is summarised in this paper^18^ sought to determine the effectiveness of interventions aimed at improving appropriate polypharmacy in older people. A recent Cochrane publication, which consisted of an overview of systematic reviews, highlighted that few reviews have considered the implications of polypharmacy on interventions seeking to improve safe and effective medicine use by consumers, including patients and their carers.^19^

## Methods

This systematic review followed the Cochrane Collaboration methodology, and is available from the Cochrane Library.^18^

### Inclusion criteria

This review looked at interventions in any setting that targeted older people (≥65 years) who had more than one long-term medical condition and were receiving polypharmacy (≥4 regular medications).

Randomised controlled trials (RCTs), including cluster RCTs (cRCTs), non-randomised controlled clinical trials, controlled before-and-after studies (CBAs) and interrupted time series (ITS) studies meeting the Effective Practice and Organisation of Care (EPOC) specification^20^ were eligible for inclusion. Any type of intervention that aimed to improve appropriate polypharmacy in any healthcare setting was eligible for inclusion. With the exception of ITS design, studies had to compare the intervention against usual care as defined by the study. Interventions studies that focused on people with single long-term conditions or who were receiving short-term polypharmacy, for example, chemotherapy, were excluded. No language restrictions were applied.

### Outcome measures

Primary outcomes were the change in the prevalence of appropriate polypharmacy and the number of hospital admissions. As there is no universally applicable tool to assess polypharmacy appropriateness in older people, validated measures of inappropriate prescribing (eg, Beers’ criteria^21^ and the Medication Appropriateness Index (MAI)^22^) were used as surrogate markers. Studies using expert opinion alone to determine medication appropriateness were excluded.

The following secondary outcomes were included: medication-related problems (eg, adverse drug reactions, medication errors); medication adherence; health-related quality of life (assessed by a validated method).

### Search methods for identification of studies

Search strategies (see full review^18^) comprised keywords and controlled vocabulary such as MeSH (medical subject headings). The following electronic databases were searched for primary studies (all records through to November 2013): Evidence-Based Medicine Reviews, Cochrane Central Register of Controlled Trials, Ovid SP, Health Technology Assessment, National Health Service Economic Evaluation Database, Cochrane Methodology Register, American College of Physicians Journal Club, the Joanna Briggs Institute, MEDLINE, EMBASE, CINAHL, EBSCO Host, PsycINFO.

Related systematic reviews were identified through the Cochrane Database of Systematic Reviews and Database of Abstracts of Reviews of Effects. Authors were contacted for further information where necessary.

### Data screening and extraction

The retrieved titles and abstracts were screened independently by two authors against inclusion criteria. Where uncertainty occurred, full-text articles were retrieved and assessed. Any remaining uncertainty or disagreement was resolved by consensus through discussion with another author. Data were extracted independently by two authors.

### Assessment of risk of bias

Two authors independently assessed risk of bias using the Cochrane Collaboration's assessment tool^23^ and used GRADE (Grades of Recommendation, Assessment, Development and Evaluation) to assess the quality of the evidence for each primary outcome for which data were pooled.^24^

### Data analysis

Intervention effect was measured using validated assessment tools of prescribing appropriateness (eg, summated MAI, Beers’ criteria). The mean and SD were calculated for summated MAI and number of Beers’ drugs postintervention in each study's intervention and control groups. Where available, the mean change (and SD) from pre to post was determined in the intervention and control group. Based on these numbers, the mean differences were calculated and results presented with 95% CIs. Estimates for dichotomous outcomes from individual studies are presented as risk ratios with 95% CIs.

If at least two studies were homogeneous in terms of participants, interventions and outcomes, the results were pooled in a meta-analysis. In the presence of statistical heterogeneity (I^2^ statistic >50%), a random-effects model was applied for meta-analysis. In the absence of statistical heterogeneity, a fixed-effects model was used.

Sensitivity analyses were conducted for studies with a high risk of bias or a unit of analysis error. Where outcome data could not be combined, a narrative summary was reported. Reporting bias was examined using risk of bias tables and funnel plots corresponding to meta-analysis of the primary outcome to assess potential publication bias. Data analysis was conducted using RevMan V.5.2.

## Results

### Results of the search

**Figure 1:**
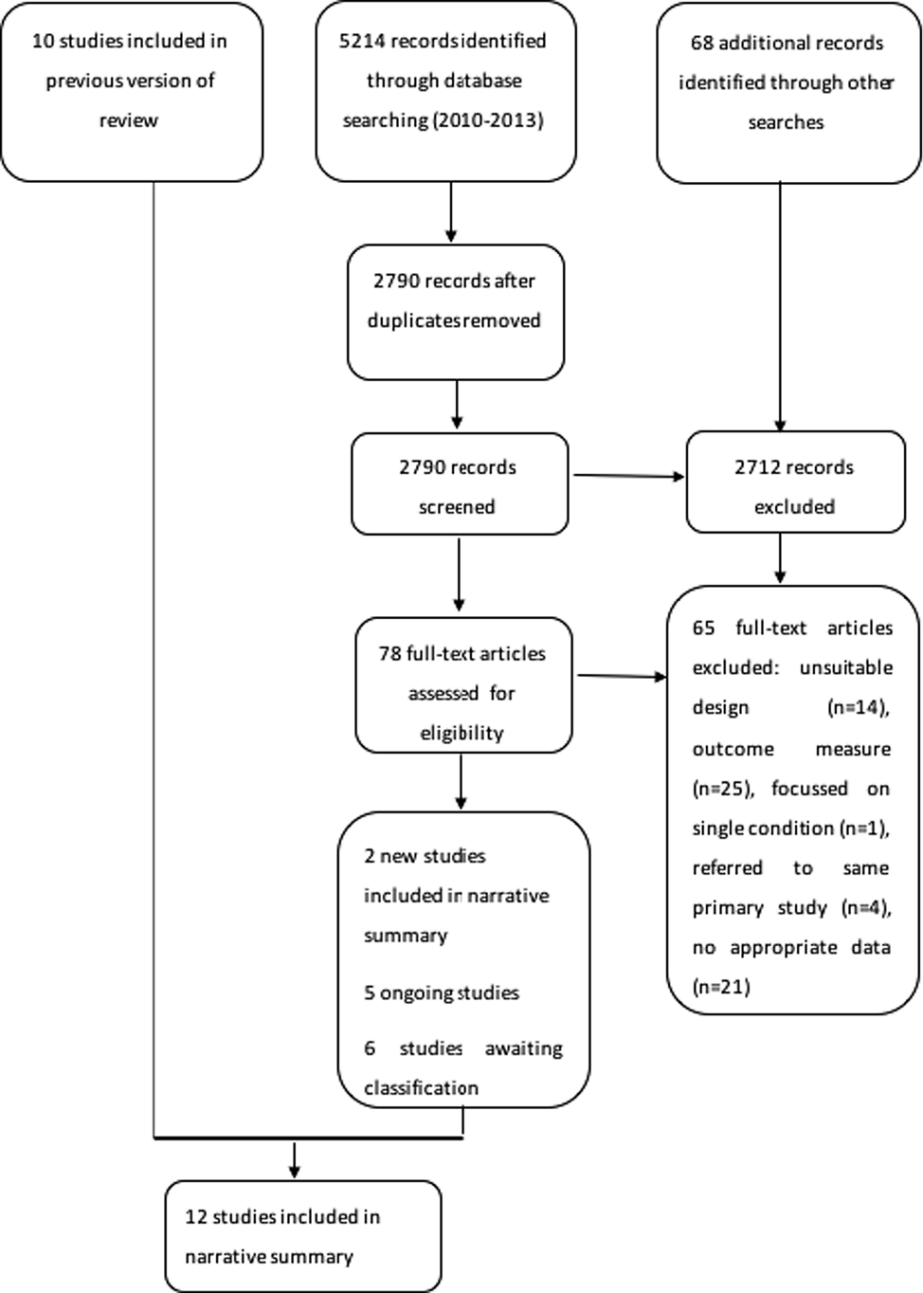
PRISMA flow chart: risk of bias in included studies (n=12).

Figure 1 provides an overview of the search. In this update, two studies were identified and added,^25^ ^26^ bringing the total number of included studies to 12. It was not possible to include data from these two studies in any meta-analysis because data were skewed or participants were not considered to be homogeneous with other study populations.

The included studies consisted of eight RCTs,^25–32^ two cRCTs^33^ ^34^ and two CBAs.^35^ ^36^ In total, 22 438 older patients were involved, the majority of whom were female (65.6%). On average, patients were 76 years old (based on 12 studies) and receiving nine medicines at baseline (based on 11 studies).

The studies were conducted in three types of settings (table 1): hospital (outpatient clinics);^27^ ^29^ ^30^ hospital/care home interface;^28^ inpatient setting;^25^ ^26^ ^31^ primary care;^32^ ^34^ nursing homes.^33^ ^35^ ^36^ The studies were carried out in five countries: Australia (two studies), Belgium (two studies), Canada (two studies), Ireland (one study) and the USA (five studies).

**Table 1:** Characteristics of included studies

Study and design	Study participants and setting	Duration and follow-up	Intervention elements	Outcomes
Hanlon *et al*^29^ RCT	208 participants (105 intervention, 103 control), Veteran Affairs Medical Centre, USA	Duration: unclear. Follow-up: 3 and 12 months after randomisation	Medication review, therapeutic recommendations, patient education	Prescribing appropriateness (MAI), HRQoL, patients’ self-reported medication compliance and knowledge, potential ADEs, participant satisfaction
Bucci *et al*^27^ RCT	80 participants (39 intervention, 41 control), university hospital clinic, Canada	Duration: unclear Follow-up: 1 month	Medication review, therapeutic recommendations, provision of medication-related information	Prescribing appropriateness (MAI), rating of pharmaceutical care activities (Purdue Pharmacist Directive Guidance score)
Tamblyn *et al*^34^ RCT	107 primary care physicians, Canada	Duration: 13 months Follow-up: terminated after an inappropriate prescription had been initiated or discontinued	Computerised decision support; computer system alerted prescribers of 159 clinically relevant prescribing problems among the elderly (McLeod criteria), the nature of the problem, possible consequences and suggested alternative therapy	Initiation and discontinuation rates of 159 prescription-related problems (McLeod criteria)
Taylor *et al*^32^ RCT	69 participants (33 intervention, 36 control), community-based family medicine clinics, USA	Duration: 12 months Follow-up: 12 months	Medication review, therapeutic recommendations, therapeutic monitoring, education of patients and healthcare professionals	Prescribing appropriateness (MAI), hospitalisations and emergency department visits, medication misadventures, medication compliance, quality of life
Crotty *et al*^33^ cRCT	154 participants (100 intervention and internal control, 54 external control), high-level residential aged care facilities, Australia	Duration: 2 case conferences 6 to 12 weeks apart Follow-up: 3 months	Medication review, multidisciplinary case conference, development of a problem list	Prescribing appropriateness (MAI), residents’ behaviour (Nursing Home Behaviour Problem Scale), monthly drug costs
Crotty *et al*^28^ RCT	110 participants (56 intervention, 54 control), hospital/long-term residential care facility interface, Australia	Duration: unclear Follow-up: 8 weeks	Transfer of medication-related information to care providers in long-term care facilities, evidence-based medication review, case conference	Prescribing appropriateness (MAI), hospital usage (unplanned visits to the emergency department and hospital readmissions), ADEs, falls, worsening of mobility behaviours, pain and increasing confusion
Schmader *et al*^30^ RCT	834 participants (430 intervention, 404 control), Veterans Affairs hospitals, USA	Duration: 12 months Follow-up: 12 months after randomisation	Medication review, therapeutic evaluation and management protocols	Prescribing appropriateness (MAI, Beers’ list), adverse drug reactions, serious adverse drug reactions, polypharmacy, medication under use
Trygstad *et al*^36^ CBA	Medicaid-dependent nursing home residents, USA	Duration: 6 months Follow-up: 3 months	Medication review, therapeutic recommendations	Prescribing appropriateness (Beers’ list), number of PAL alerts, potential medication problems
Spinewine *et al*^31^ RCT	186 participants (96 intervention, 90 controls), university teaching hospital, Belgium	Duration: from admission to discharge Follow-up: 1, 3 and 12 months	Medication review, pharmaceutical care plan, therapeutic recommendations, information provision to healthcare professionals, patient/carer education, communication with GP	Prescribing appropriateness (MAI, Beers’ list, ACOVE), mortality, hospitalisation (readmission or visit to an emergency department), medication use (including unnecessary drug use), satisfaction with information provided at admission and discharge
Trygstad *et al*^35^ CBA	Medicaid-dependent nursing home residents, USA	Duration: 3 months Follow-up: 3 months	Medication reviews, computerised prescribing alerts, therapeutic recommendations	Prescribing appropriateness (Beers’ list), number of PAL alerts, potential medication problems
Gallagher *et al*^25^ RCT	382 participants (190 intervention, 192 control), university hospital, Ireland	Duration: unclear Follow-up: 2, 4 and 6 months post discharge	Medication review, discussion with attending medical team, follow-up written communication, recommendations (STOPP/START), communication of medication changes to GPs using discharge summary	Prescribing appropriateness (MAI and AUM), mortality, hospital readmissions, falls, frequency of general practitioner visits
Dalleur *et al*^26^ RCT	146 participants (74 intervention, 72 control), university teaching hospital, Belgium	Duration: unclear Follow-up: at discharge and 1 year after discharge	Medication review, therapeutic recommendations, standard IGCT care	Discontinuation of potentially inappropriate medications (STOPP criteria), clinical significance of prescribing recommendations (STOPP criteria)

ACOVE, Assessing Care of Vulnerable Elderly; ADE, adverse drug event; AUM, Assessment of Underutilisation of Medication; CBA, controlled before-and-after studies; cRCT, cluster randomised controlled trial; GP, general practitioner; HRQoL, health-related quality of life; IGCT, inpatient geriatric consultation team; MAI, Medication Appropriateness Index; PAL, Prescription Advantage List; RCT, randomised controlled trial; START, Screening Tool to Alert doctors to Right Treatment; STOPP, Screening Tool of Older Person's Prescriptions.

### Description of interventions

All interventions were classified as organisational according to EPOC definitions.

Eleven studies examined complex, multifaceted, pharmaceutical care-based interventions in various settings, using validated assessment criteria to give recommendations on improving the appropriateness of prescribing. In all settings, pharmaceutical care (ie, responsible provision of drug therapy to achieve definitive outcomes that improve patients’ quality of life^37^) was commonly provided by pharmacists working closely with other HCPs.

The models of pharmaceutical care provided were complex and variable. For example, pharmacists conducted independent medication reviews either using patient notes^28^ ^33^ or with patients during a face-to-face encounter.^27^ ^29–32^ ^34^ In other cases, recommendations from medication reviews were followed up with prescribers and other HCPs.^27–29^ ^31^ ^33^

Patient education was provided as part of the intervention in four studies involving face-to-face interventions. Patients were given information about their prescribed medications (eg, administration) and specialised medication scheduling tools (eg, monitored dosage systems) to encourage adherence.^27^ ^29^ ^31^ ^32^

Education was also provided to prescribers and other HCPs involved in the multidisciplinary team as part of the intervention in five studies.^27–29^ ^31^ ^33^

The only unifaceted study^34^ examined computerised decision support (CDS) provided to general practitioners in their own practices.

The timing of intervention provision was variable. A number of interventions were delivered at specific time points, for example, hospital admission, attendance at outpatient clinics,^27^ ^29^ ^30^ ^32^ nursing home visits,^33^ ^35^ ^36^ hospital discharge to a nursing home.^28^ In other cases, interventions were delivered over a period of time, such as during hospital inpatient stay and at discharge.^30^ ^31^

### Risk of bias in included studies

**Figure 2:**
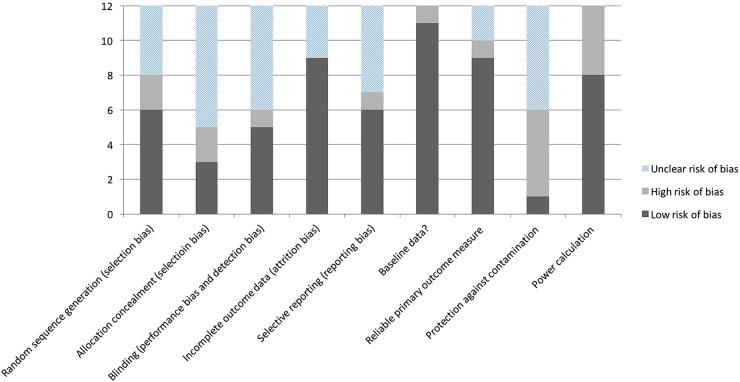
Risk of bias in included studies (n=12).

The included studies showed evidence of potential bias (figure 2). Only three studies showed evidence of allocation concealment^25^ ^28^ ^33^ and only one study demonstrated protection against contamination.^33^

Funnel plots of postintervention estimates of the change in MAI and summated MAI showed little evidence of publication bias.^18^

### GRADE approach to quality assessment

Based on GRADE,^24^ the overall quality of evidence for each primary outcome for which data were included in a meta-analysis was rated as ‘low’ or ‘very low’ (table 2). Although all studies included in the meta-analyses involved randomisation, and, where assessed, no evidence of publication bias was found,^18^ the quality of evidence was downgraded for each outcome based on other GRADE considerations (ie, study limitations, consistency of effect, imprecision, indirectness).

**Table 2:** Summary of findings table

			Effect estimate			
Outcome	Number of studies	Number of participants	Usual care	Pharmaceutical care	Quality of the evidence (GRADE approach)	Comments
Summated MAI score (postintervention)	5 (27–31)	965	Mean summated MAI score ranged across control groups from 6.5 to 19.3	Mean summated MAI score in the intervention groups was 3.88 lower (5.4 to 2.35 lower)	Low*†	
Change in MAI score (from baseline to follow-up)	4 (27 28 31 33)	424	Mean change in MAI score ranged across control groups from 0.41 to 2.86	Mean change in MAI score in the intervention groups was 6.78 lower (12.34 to 1.22 lower)	Very low*†‡§	A sensitivity analysis showed that the mean change in MAI score in the intervention group was 1.79 lower (3.73 lower to 0.16 higher)¶
Number of Beers drugs per patient (post-intervention)	2 (30 31)	586	Mean number of Beers drugs per participant ranged across control groups from 0.04 to 0.4	Mean number of Beers drugs per participant in the intervention groups was 0.1 lower (0.28 lower to 0.09 higher)	Very low*‡§	

GRADE Working Group grades of evidence.

High quality: Further research is very unlikely to change our confidence in the estimate of effect.

Moderate quality: Further research is likely to have an important impact on our confidence in the estimate of effect and may change the estimate.

Low quality: Further research is very likely to have an important impact on our confidence in the estimate of effect and is likely to change the estimate.

Very low quality: We are very uncertain about the estimate.

*Limitations in the design of studies included in the analysis such as lack of protection against contamination and lack of allocation concealment resulted in downgrading of the quality of evidence.

†A validated assessment of underprescribing was not included in all studies; therefore, the findings answered a restricted version of the research question. This resulted in downgrading of the quality of evidence.

‡Statistically significant heterogeneity, variation in effect estimates and non-overlapping CIs between studies resulted in downgrading of the quality of evidence.

§Imprecision in effect estimates was observed whereby CIs were wide and/or crossed the line of no effect.

¶Two studies were excluded from the analysis because of a unit of analysis error^33^ and an outlying effect estimate with a high risk of bias.^31^

GRADE, Grades of Recommendation, Assessment, Development and Evaluation; MAI, Medication Appropriateness Index.

### Prevalence of appropriate use of polypharmacy

The primary outcome of interest was the change in the prevalence of appropriate polypharmacy. Seven validated measures of prescribing appropriateness were used in the included studies, either alone or in combination.

#### Medication Appropriateness Index

The MAI was used in three ways to assess the appropriateness of polypharmacy. First, data from four studies (210 intervention participants, 214 control participants) were pooled in a meta-analysis using the change in summated MAI score from baseline to follow-up.^27^ ^28^ ^31^ ^33^ There was a greater overall reduction in the mean change in summated MAI score in the intervention group compared with the control (mean difference −6.78, 95% CI −12.34 to −1.22; table 2). There was marked heterogeneity between the studies (I^2^=96%, p<0.0001). Sensitivity analyses in which one study with a unit of analysis error (nursing homes were the unit of randomisation but the analysis was conducted at patient level)^33^ and another study with a large effect size and high risks of bias^31^ were removed from analysis showed consistent changes in summated MAI with variable effects on heterogeneity (table 2).

Second, postintervention pooled data from five studies^27–31^ (488 intervention participants, 477 control participants) showed a lower summated MAI score (mean difference −3.88, 95% CI −5.40 to −2.35) in the intervention group compared with the control group (table 2). There was little evidence of heterogeneity between these estimates (I^2^=0%). This was consistent with the findings of Gallagher *et al*,^25^ which were not included in the meta-analysis because the data were skewed.

Third, one study^32^ expressed the MAI score as the number of inappropriate prescriptions. The percentage of inappropriate prescriptions decreased in all MAI domains (n=10) in the intervention group and increased in five domains in the control group. These data could not be included in a meta-analysis.

#### Beers’ criteria

Pooled data from two studies^30^ ^31^ (298 intervention participants, 288 control participants) showed that intervention group participants were prescribed fewer Beers’ drugs than control group participants postintervention (mean difference −0.1, 95% CI −0.28 to 0.09; I^2^=89%; table 2).

Spinewine *et al*^31^ also reported the proportion of patients taking one or more Beers’ drugs preintervention and postintervention. Similar improvements were reported in the proportion of intervention and control group patients receiving one or more Beers’ drugs between hospital admission and discharge (OR 0.6, 95% CI 0.3 to 1.1). As this was the only study to report the results in this format, meta-analysis was not possible.

#### McLeod criteria

One study used the McLeod criteria^38^ to identify the initiation and discontinuation rates of 159 prescription-related problems.^34^ The reported relative rate of initiation of inappropriate prescriptions for the intervention group was 0.82 (95% CI 0.69 to 0.98). However, the intervention did not appear to have an effect on the relative rate of discontinuation of pre-existing prescription-related problems (1.06, 95% CI 0.89 to 1.26). Meta-analysis was not possible as these criteria were not used in other studies.

#### STOPP and START criteria

Two studies^25^ ^26^ used the Screening Tool of Older Person's Prescriptions (STOPP) criteria to screen for PIP in older patients admitted to hospital. Gallagher *et al*^25^ reported lower (p<0.001) proportions of patients in the intervention group compared with the control group with one or more STOPP criteria medications for each of the postintervention assessments (discharge, 2, 4 and 6 months postdischarge). Dalleur *et al*^26^ reported no difference in the proportion of patients with one or more STOPP criteria medications from hospital admission to discharge between the intervention and control groups (OR 1.5, 95% CI 0.49 to 4.89, p=0.454). However, at group level, the discontinuation rate of potentially inappropriate medications as identified using STOPP criteria was higher in the intervention group compared with the control group (OR 2.75, 95% CI 1.22 to 6.24, p=0.013). Data from these studies were not pooled because participants were not homogeneous.

In the Gallagher *et al*^25^ study, the Screening Tool to Alert doctors to Right Treatment (START) criteria were also used. For each of the postintervention assessments (discharge, 2, 4 and 6 months postdischarge), lower proportions of patients with one or more START criteria medications were reported in the intervention group compared with the control group (p<0.001). This was the only study that used these criteria; therefore, meta-analysis was not possible.

#### Assessment of Underutilisation of Medication

Two studies assessed under-use of medication using the Assessment of Underutilisation of Medication (AUM) index.^25^ ^30^ Gallagher *et al*^25^ reported a greater reduction in the proportion of intervention group patients with prescribing omissions postintervention (by the AUM index) compared with the control group (absolute risk reduction 21.2%, 95% CI 13.3% to 29.1%). Schmader *et al*^30^ reported a reduction in the number of conditions with omitted drugs postintervention in the intervention group relative to the control group; the difference in change in AUM score was −0.3 (p<0.0001). As each study assessed underprescribing on two different levels (ie, patient, medical condition), meta-analysis was not possible.

#### ACOVE

Spinewine *et al*^31^ reported that the magnitude of the reduction in Assessing Care of Vulnerable Elderly (ACOVE) scores was greater in the intervention group (baseline score: 50.0, postintervention score: 14.6, p<0.001) compared with the control group (baseline score: 58.9, postintervention score: 44.4, p=0.02). Intervention patients were six times more likely than control patients to have at least one prescribing improvement based on these criteria (OR 6.1, 95% CI 2.2 to 17.0). Meta-analysis was not possible; no other studies used this outcome measure.

### Hospital admissions

Five studies measured hospital admissions.^25^ ^28^ ^31^ ^32^ ^35^ Two studies^25^ ^31^ reported no difference in hospitalisations between intervention and control groups at follow-up and the remaining studies reported some overall reductions in hospital admissions between the two groups. The statistical significance of these reductions varied based on the methods of assessment employed in the individual studies. Owing to differences in the measurement of hospital admissions and the expression of results, meta-analysis was not possible.

### Secondary outcomes

Meta-analysis of secondary outcome assessments was not possible due to differences across studies in design and reporting. Evidence of the effect of the interventions on medication-related problems (six studies)^28–30^ ^32^ ^35^ ^36^ was conflicting. One study reported improved adherence scores in intervention patients.^32^ No differences in HRQoL were reported between intervention and control groups at baseline or follow-up (two studies).^29^ ^32^

## Discussion

Given the association between polypharmacy and PIP in older people,^11^ ^17^ interventions to improve appropriate polypharmacy in this cohort are of considerable importance. Only two studies were added to the original review, bringing the total number of studies included in the updated review to 12. These two additional studies did not change the conclusions of the original review and serve to highlight the lack of intervention studies aimed at improving appropriate polypharmacy in older people that have been conducted to date. Coupled with the findings of Ryan *et al*,^19^ it is evident that interventions targeting polypharmacy are under-researched at both the level of healthcare provider and recipient.

The included studies aimed to ensure the prescribing of appropriate medications to older people that enhanced their quality of life. However, several studies focused on reducing the number of prescribed medications without assessing underprescribing and, therefore, did not consider the overall appropriateness of prescribing. This needs to be addressed as underprescribing is common in older populations with variable prevalence rates depending on medication classes and care settings.^39^ Nevertheless, the interventions reduced inappropriate prescribing with resultant improvements in the appropriateness of polypharmacy in older patients. For example, pooled data showed a significant reduction in intervention group patients’ mean MAI score compared with control group patients (table 2). Assessments involving other validated tools also showed improvements in the appropriateness of prescribing. Although these results are promising and indicate that the interventions described in this review were successful in improving appropriate polypharmacy, the clinical impact is not known. For example, it is unclear to what extent a reduction in the magnitude of 3.88 in summated MAI score (a weighted average rating based on 10 assessment criteria) represents a clinically significant reduction in the risk of harm (table 2). This is because the predictive validity of many tools that are currently used to evaluate prescribing appropriateness has not been established.^40^ Therefore, the impact of improvements on the overall appropriateness of prescribing on clinical outcomes is unclear.

The findings from our review are consistent with other reviews for a number of outcomes. For example, a related Cochrane review of interventions to optimise prescribing for older people in care homes^41^ found no evidence of an intervention effect on ADEs and hospital admissions. Other studies of interventions conducted across various settings have also been unable to detect the effect of pharmaceutical care on these outcomes.^42^ ^43^

Despite the uncertainty as to the effect of the identified interventions to improve appropriate polypharmacy on a number of outcome measures, this review provides useful guidance for the direction of future research.

### Strengths and weakness of this review

The updated Cochrane systematic review that is summarised in this paper represents the most comprehensive overview, using a rigorous methodology, of the existing body of evidence of the effectiveness of interventions aimed at improving appropriate polypharmacy in older patients. Previous reviews have assessed interventions targeting medication use in older people, but have not focused on polypharmacy or exclusively used validated assessment tools.^7^ ^44^ No language restrictions were placed on the search strategy and all of the studies were published in English, including those studies that were conducted in countries where English is not the first language. Despite the small number of included studies, no apparent publication bias was detected.

Overall, the included studies were limited by their small sample sizes and poor quality, with little opportunity to pool data. There was evidence of potential biases (figure 2) in the studies which may have influenced the reported effect estimates. Although improvements in appropriate polypharmacy were noted, the findings of meta-analyses relating to MAI scores should be treated cautiously, as the intervention did not seem to work consistently across all studies.

It must also be noted that assessments were based on surrogate markers and the clinical significance of these improvements in terms of clinically relevant outcomes, for example, hospital admissions, is unclear as meta-analysis was not possible. Several studies focused on reducing the number of medications, rather than improving the overall appropriateness of prescribing, including underprescribing.

### Implications for clinical practice and future research

Inappropriate prescribing is highly prevalent and commonly associated with polypharmacy in older populations.^11^ ^17^ However, rigorous evaluations of interventions seeking to address this are lacking. The findings of this review indicate that pharmaceutical care-based interventions appear to improve appropriate polypharmacy in older people based on observed reductions in inappropriate prescribing, especially when the provision of care involves a multidisciplinary element.^25^ ^27–33^ CDS showed potential as an intervention, although this was evaluated in only one study.^34^

Surrogate markers of appropriate polypharmacy were used as there is no universally applicable tool to assess the appropriateness of polypharmacy. Despite observed improvements in prescribing appropriateness, it is unclear if the identified interventions resulted in clinically significant improvements, for example, reduction in medication-related problems. In addition to the above noted issues with the predictive validity of existing tools for assessing appropriate prescribing, many studies did not assess outcomes such as adherence, hospitalisations and quality of life, which are arguably the critical outcomes for patients and some studies may have lacked sufficient follow-up periods to detect any significant changes. Future studies should focus on these types of clinical outcomes.

Overall, the quality and reporting of included studies was poor. Future research should pay greater attention to available guidance on intervention development and evaluations^45^ to ensure rigour in study design. Methods of specifying and reporting complex interventions,^46^ as well as their implementation strategies, are necessary to strengthen the evidence base required for interventions to be more effective, implementable and replicable across different settings.^47^ ^48^

Future studies should use clearer definitions of appropriate polypharmacy because the term ‘polypharmacy’ can be both negative and positive, and this duality of meaning makes objective research difficult.^49^ A recent report by the King's Fund in the UK^6^ raised the need to reconsider current definitions of polypharmacy due to the increasing numbers of medications being prescribed to patients. The publication of this report^6^ coincided with the abstract screening process in the update of this review. Therefore, for the purpose of this update, the definition of polypharmacy was not changed from the original review. However, future updates may need to reconsider the criteria used to define polypharmacy.

Development of new, universal, easily applied, valid and reliable outcome measures to evaluate effectiveness of interventions should be a priority for future research. Ideally the measure should be globally applicable across various healthcare and cultural settings; for example, STOPP/START are validated instruments that could help to fulfil this need.^50^ In contrast to other tools, such as the Beers’ criteria, STOPP/START have been specifically developed for use in European countries. Although STOPP/START-related research is still at a relatively early stage, the criteria are endorsed by the European Union Geriatric Medicine Society and set for wider application in future research.^51^ The use of START offers a promising strategy to decrease underprescribing^39^ and could serve to improve appropriate polypharmacy when combined with STOPP.

## Conclusions

The findings of an updated Cochrane review that are summarised in this paper highlight the lack of existing intervention studies of suitable quality aimed at improving the appropriate use of polypharmacy in older patients. Overall, the interventions included in this review demonstrated benefits in this respect based on observed reductions in inappropriate prescribing. However, it remains unclear if interventions resulted in clinically significant improvements in terms of hospital admissions, medication-related problems and patients’ overall quality of life. Future studies would benefit from guidance relating to intervention development, evaluation and reporting. In addition, more detailed and systematic reporting of interventions in published papers could facilitate replication of effective interventions and uptake into clinical practice.
